# Endocrine Dysfunction Following Bariatric Surgery: A Systematic Review of Postoperative Changes in Major Endocrine Hormones

**DOI:** 10.7759/cureus.77756

**Published:** 2025-01-21

**Authors:** Ammar Shahid Tanweer, Majd H Shaheen, Bashayer A Alshamsi, Mahra A Almazrouei, Rama M Almasri, Ariba Shahid Tanveer, Jana M Rajeh

**Affiliations:** 1 Internal Medicine, RAK Medical and Health Sciences University, Ras Al Khaimah, ARE; 2 Pediatrics, Fujairah Hospital, Fujairah, ARE

**Keywords:** bariatric surgery, endocrine dysfunction, gonadal hormones, pancreatic endocrine hormones, postoperative changes, thyroid function

## Abstract

Bariatric surgery (BS) is an effective intervention for obesity and related metabolic disorders, significantly improving metabolic health and alleviating hormonal imbalances. However, it induces complex endocrine changes that can lead to dysfunctions, impacting the somatotropic, gonadal, thyroid, pancreatic, and adrenal axes. This review highlights the dual effects of BS on the endocrine system.

A comprehensive review of peer-reviewed studies using PRISMA guidelines was conducted, focusing on human research evaluating pre and postoperative endocrine parameters. Studies were selected for their relevance and quality in elucidating the endocrine consequences of BS. BS restores growth hormone secretion and improves fertility but may disrupt insulin-like growth factor-1 recovery and sex hormone balance, leading to bone loss and catabolic states. Postprandial insulin hypersecretion can result in hyperinsulinemic hypoglycemia, with impaired counter-regulatory hormone responses. Secondary hyperparathyroidism and reduced bone density highlight additional risks. Changes in thyroid hormone levels have implications for both hypothyroid and euthyroid patients. These findings underscore the interplay between improved metabolic control and potential endocrine dysfunctions.

The current evidence predominantly comprises association studies that may not be of quality for safe clinical decision-making, highlighting the need for high-quality research to establish causality and refine therapeutic strategies. Bridging knowledge gaps in the mechanisms underlying these changes is crucial to optimizing BS outcomes. A holistic approach integrating preoperative screening, individualized postoperative care, and targeted therapies is essential to mitigate complications while maximizing benefits.

## Introduction and background

The global prevalence of overweight and obesity has risen to critical levels, presenting major challenges for health systems worldwide. These conditions are marked by an excessive or abnormal accumulation of body fat, which can detrimentally affect an individual’s health and overall quality of life [1]. Body mass index (BMI) is a commonly utilized tool to classify overweight and obesity, calculated by dividing a person’s weight in kilograms by the square of their height in meters (kg/m²). In adults, a BMI of 18.5-24.9 kg/m² is considered healthy, while a BMI of 25 kg/m² or higher is categorized as overweight, and 30 kg/m² or above is classified as obesity. In Asian and South Asian populations, overweight is defined as a BMI between 23 and 24.9 kg/m², while obesity is classified as a BMI greater than 25 kg/m² [2]. The prevalence of obesity has alarmingly increased over the last four decades, impacting both the young and the elderly [3]. Obesity is a major risk factor for and contributor to increased morbidity and mortality, mainly from cardiovascular disease (CVD) and diabetes, but also from cancer and other acute and chronic diseases like osteoarthritis, liver and kidney disease, sleep apnea, and depression, as evidenced by large, high-quality longitudinal or prospective studies. Weight loss can significantly lower the risk for most of these comorbid diseases [4]. 

Due to its ability to significantly reduce weight and improve related comorbidities, bariatric surgery (BS) has become one of the promising treatment options for people who are extremely obese [5]. BS is the most successful long-term therapy for severe obesity that is complicated by T2DM [6]. According to the 2022 American Society for Metabolic and Bariatric Surgery (ASMBS) and International Federation for the Surgery of Obesity and Metabolic Disorders (IFSO) guidelines, metabolic and bariatric surgery (MBS) is recommended for individuals with a body mass index (BMI) ≥35 kg/m², regardless of the presence, absence, or severity of co-morbidities, and in Asian populations for individuals with a BMI ≥27.5 kg/m² [7]. It should also be considered for individuals with metabolic disease and a BMI of 30-34.9 kg/m² [7]. There is growing evidence that, in cases where T2DM is being treated optimally medically, patients with a BMI of 30.0 to 34.9 kg/m² and T2DM should also be evaluated for BS if their hyperglycemia is not effectively controlled [8].

It has been shown that BS is a successful treatment for achieving both long-term weight loss and a substantial metabolic improvement that goes beyond weight loss alone [9]. Because of its well-established positive effects on metabolic characteristics, several researchers have suggested that BS could be used as a metabolic surgery to treat moderate obesity [9]. Furthermore, in addition to affecting metabolic balance, BS has significant effects on endocrine systems that are not solely involved in metabolic function. It has also been demonstrated that the various BS procedures have an impact on bone health and the somatotropic, corticotropic, and gonadal axes, with the majority being beneficial [9]. Candidates for metabolic and bariatric surgery (MBS) should undergo evaluation by a multidisciplinary team with expertise in medical, surgical, psychiatric, and nutritional care. Such comprehensive assessments have been consistently emphasized, recognizing the complexity of obesity and the need for a thorough risk-benefit analysis. This approach also helps patients understand the lifelong changes required after surgery, benefiting from the insights and guidance of various healthcare professionals [7]. A multidisciplinary team plays a key role in evaluating and addressing a patient’s modifiable risk factors to minimize perioperative complications and enhance overall outcomes. However, the final decision regarding surgical readiness should ultimately be made by the surgeon [7].

Despite the well-documented benefits of BS, there is a notable gap in the literature regarding its potential adverse effects on the endocrine system. This review aims to address this gap by systematically exploring the postoperative endocrine dysfunctions associated with various bariatric procedures. Specifically, it seeks to investigate the impact of BS on thyroid, parathyroid, pancreatic, sex hormone, and pituitary functions, emphasizing the need for clinical studies to establish causal relationships and better understand these complications. With approximately 279,000 BS performed in the United States in 2022 alone [10], the need for further research in this area is both urgent and critical to understanding and mitigating these complications.

## Review

Methodology

Search Strategy

**Figure 1:**
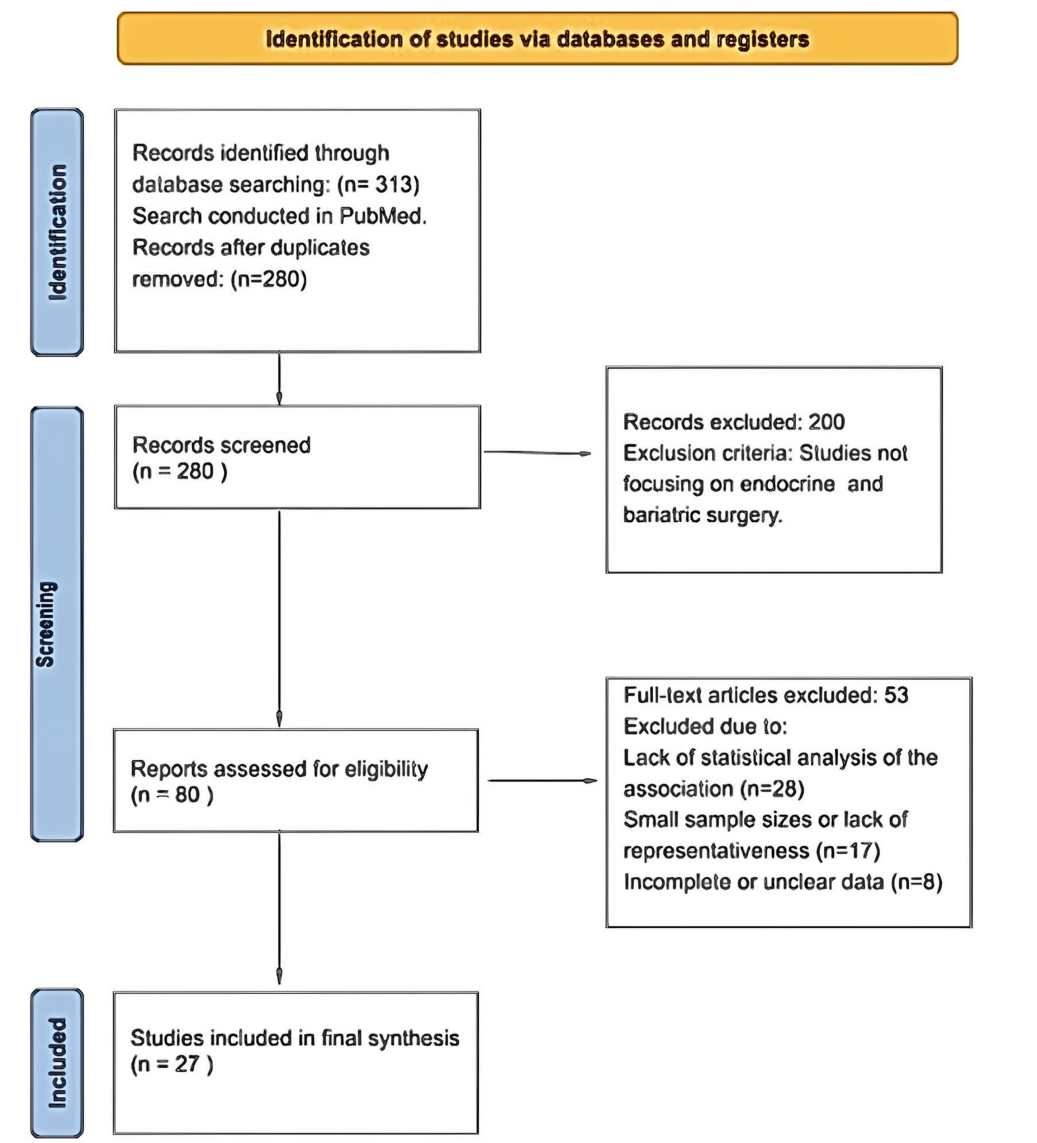
PRISMA flowchart of selected articles. PRISMA: Preferred Reporting Items for Systematic Reviews and Meta-Analyses.

We conducted a systematic review in accordance with the PRISMA (Preferred Reporting Items for Systematic Reviews and Meta-Analyses) guidelines to evaluate the endocrine effects of BS. A comprehensive search was performed using databases including PubMed. Keywords such as “bariatric surgery and endocrine changes”, “BS and thyroid dysfunction”, and “BS and metabolic axes” were employed, combined with Boolean operators (AND/OR) to refine search results. Initial searches identified 313 articles, and after removing duplicates (n=33), 280 titles and abstracts were screened. A total of 80 articles were assessed for eligibility, and 27 were included in the final synthesis. The study selection process is illustrated in the PRISMA flowchart (Figure 1).

Inclusion and Exclusion Criteria

Inclusion criteria targeted studies published between 2000 and 2024 that investigated pre and post-operative endocrine parameters in bariatric surgery patients. Eligible studies included observational studies (cohort, cross-sectional) and clinical trials that explored the impacts of BS on growth hormone, thyroid, gonadal, pancreatic, and adrenal axes. Excluded were case reports, reviews without primary data (n=28), qualitative studies (n=10), and articles lacking statistical analyses (n=8). Studies with small sample sizes or incomplete data were also excluded (n=7).

Results

From the initial search of 313 articles, 280 remained after duplicate removal, and 80 full-text articles were reviewed. Following the application of inclusion and exclusion criteria, 27 studies were included. The detailed process is summarized in the PRISMA flowchart (Figure 1). These studies highlighted the dual effects of bariatric surgery, such as improvements in growth hormone secretion and fertility, but also risks like postprandial hyperinsulinemic hypoglycemia and secondary hyperparathyroidism. These findings underscore the complexity of endocrine changes post-BS, with implications for long-term management and patient selection criteria.

Review

Somatotropic Axis: Growth Hormone-Releasing Hormone​​​, Growth Hormone, Insulin-Like Growth Factor 1, and Somatostatin

The anterior pituitary of the brain produces human growth hormone (HGH). The main regulatory factors include growth hormone-releasing hormone (GHRH), produced in the hypothalamus; somatostatin, produced in the pancreas, gastrointestinal tract, and hypothalamus; and ghrelin, produced in the gastrointestinal tract [11]. HGH acts directly by attaching to target cells and indirectly by the release of insulin-like growth factor 1 (IGF-1) by hepatocytes [11].

An acquired functional decrease in GH production and GH response to stimuli (such as insulin-induced hypoglycemia, arginine, arginine-GHRH, sleep, or exercise) is linked to obesity; this decrease is reversible following substantial weight loss [12,13]. Similarly, obesity has been linked to a decrease in GH half-life by increasing GH clearance and acting as a negative determinant for GH secretion frequency and width [14]. Hormonal GHRH, somatostatin, or ghrelin dysregulation have been suggested as the neuroendocrine changes that underlie decreased plasma GH levels in obesity [15,16]. There have been conflicting reports on serum IGF-1 levels in obese individuals, with both normal [14,17,18] and lower IGF-1 levels reported [19]. 

Several studies report that BS restores somatotropic axis alterations in severely obese patients. For example, GH secretion significantly increases after biliopancreatic diversion (BPD) [20] and partially recovers following roux-en-Y gastric bypass (RYGB) [21]. Mittempergher et al. found that GH levels rose more after malabsorptive procedures (2.7-fold) compared to restrictive ones (1.4-fold) [14]. Britt Edén Engström et al. observed improved GH secretion and IGF-1 levels in women at 6 months and in both sexes at 12 months after RYGB [22]. These findings suggest that obesity-induced GH dysfunction is reversible after BS. But this is not always the case.

The effect of bariatric surgery on IGF-1 is not this linear. Barring a couple of discrepancies [14,23], the secretion of IGF-1 after BS is impaired for a very long time, which is attributed to the catabolic state produced by RYGB and BPD [22,24]. This effect is not unique to these surgeries alone. The same discrepancy was noted in a series of obese female patients who underwent adjustable gastric banding laparoscopically (LAGB), with a fifth of the patients having IGF-1 levels below the age-appropriate ranges [25]. There is a dissociation between the restoration of GH and IGF-1, which is probably an indicator of an unrecognized and persistent, long-term catabolic state that is produced by these surgeries. This might be of more significance than previously estimated. Weight loss and fat mass loss were reported to be lower in patients who did not normalize this GH/IGF-1 axis as compared to patients who did [25,26]. Further trials need to investigate if routine analysis of the GH/IGF-1 axis is required prior to surgery.

Oxytocin

Becetti et al. conducted a prospective study of 52 participants to analyze the serum oxytocin concentration in adolescents and young adults undergoing sleeve gastrectomy (SG) and its correlation to body composition and bone mineral density (BMD) [27]. They found that serum oxytocin was reduced 12 months postoperatively, and it correlated positively with decreases in lean mass. Since lean mass is a strong predictor of BMD [28], further studies need to evaluate these findings with a larger sample size and analyze if oxytocin therapy can be used to slow down or prevent this loss of lean mass.

Gonadal Axis: Growth Hormone-Releasing Hormone​​​​, Follicle Stimulating Hormone, Luteinizing Hormone, Testosterone, and Estradiol 

The benefits of BS in improving hypothalamic-pituitary-gonadal (HPG) axis function have been well documented. A recent systematic review and meta-analysis provided evidence that BS improves fertility outcomes in both males and females using hormone level measurement and sexual function index scores [29]. Other reviews have highlighted that BS leads to improvements in sex hormones in women, which are linked to better menstrual cycles, enhanced fertility, and changes in hair and fat distribution [30]. Additionally, these changes are associated with a reduced risk of endometrial cancer and improvements in the hormonal and metabolic abnormalities characteristic of polycystic ovary syndrome (PCOS) [31,32].

Although these changes are mostly positive, changes in sex hormones are linked to detrimental effects on the bones. A prospective study conducted by Nimmala et al. in young patients undergoing SG found that bone loss following SG was associated with changes in sex steroids [33]. Another study over a longer 24-month period concluded the same findings [34]. Yang et al. performed a retrospective study that found that changes in total estrogen post-SG were significantly associated with total hip BMD loss in women but not men, while changes in sex-hormone binding globulin (SHBG) were significantly associated with total hip BMD in men [35]. These changes in sex hormones and their detrimental effects on bone parameters following BS are highlighted in Table 1.

**Table 1:** Changes in bone parameters and sex hormones following bariatric surgery. SG: Sleeve gastrectomy; BMD: Bone mass density; SHGB: Sex hormones binding globulin.

Study	Study design	Population	Number of participants	Methods	Key findings
Nimmala et al. [33]	Longitudinal observational study	Youth aged 13-25 years undergoing SG	64	Analysis of sex hormones, bone turnover markers, and BMD, among other bone parameters, over 12 months.	Among females, reductions in total hip BMD Z-scores were positively associated with changes in estrone.
Kaur et al. [34]	Longitudinal observational study	Youth aged 13-25 years undergoing SG	53	Analysis of bone turnover markers, sex hormones, sex hormone binding globulin (SHBG), and BMD, among other parameters for bone strength estimates over 24 months.	Among females, after controlling for age, decreases in estrogen were associated with decreases in spine BMD Z-scores and radial total and trabecular BMD
Yang et al. [35]	Retrospective study	Middle-aged men and non-menopausal women undergoing SG	49	Anthropometrics, bone turnover markers, calciotropic hormones, BMD, SHBG, and gonadal steroids were measured preoperatively and at 6 and 12 months postoperatively.	SHBG was significantly associated with total hip BMD loss in men (p=0.019), but not in women; total estrogen was significantly associated with total hip BMD loss in women (p=0.01) but not in men.

Other studies have tried establishing a similar relationship between changes in sex hormones and detrimental changes observed post-BS. Rutledge et al. suggested that a decrease in estrogen following gastric bypass (GB) in women might be associated with depression and anxiety out of proportion to the surgical outcome [36]. They conducted a study and found that empiric trials of topic estradiol patch post-BS improved depressive symptoms in 67% of the women [36]. Alterations of testosterone and estrogen, among other hormones following RYGB and SG, are associated with significant ongoing bone loss, which persists despite vitamin D and calcium supplementation [37]. Further studies need to be conducted in order to establish if a causal relationship exists between BS and these changes with larger sample sizes and to evaluate hormone replacement therapy as a modality to prevent such changes. 

*Hypothalamic-Pituitary-Thyroid Axis: Thyrotropin Releasing Hormone, Thyroid-Stimulating Hormone* *, T3 and T4*

Researchers have found that BS is associated with significant decreases in the levels of Thyroid-Stimulating Hormone (TSH), Free Triiodothyronine (FT3), and Triiodothyronine (T3) levels post BS and that this decrease of TSH is associated with favorable outcomes in overt and subclinical hypothyroid patients [38,39]. However, while this decrease is beneficial in hypothyroid patients, the effect of this decrease in euthyroid patients has yet to be investigated fully. Newer studies undertaken by researchers have tried to investigate this further.

Gokosmanoglu et al. conducted a prospective study on 472 patients without prior thyroid disease or dysfunction to evaluate thyroid homeostasis after BS. They observed a significant decrease in TSH levels and an increase in thyroid parenchymal echogenicity, with no significant changes in FT3 and Free Thyroxine (FT4) levels [40]. Similar findings were reported by other authors, who noted a significant decline in TSH in euthyroid patients without corresponding changes in FT3 and FT4 [41,42].

However, some studies have reported changes in thyroid hormones other than TSH following BS. Liu et al. documented a decrease in both TSH and FT4 in euthyroid patients [43], while multiple studies found significant reductions in TSH and FT3 levels [44-48]. Tian et al. observed a broader pattern of decreases, including TSH, FT4, FT3, total thyroxine (TT4), and total triiodothyronine (TT3) [49].

In contrast, Kyrou et al. reported an increase in thyroid parenchymal echogenicity in euthyroid patients post-BS but no significant changes in thyroid function tests; however, their findings were limited by a small sample size [50]. Another study observed only non-significant changes in TSH levels [51]. On the other hand, Chen et al. found a significant increase in FT4 levels after Laparoscopic Sleeve Gastrectomy (LSG) in euthyroid patients, accompanied by a decline in TSH and FT3 levels [52].

The correlation of these changes in TSH with other measured parameters is elaborated in Table 2. While these changes are statistically significant, their correlation to any clinically significant signs and symptoms in previously euthyroid patients has yet to be investigated. These studies are limited by their focus on measured values of hormones without establishing the clinical effects, if any, produced by their changes.

**Table 2:** Changes in thyroid hormones following bariatric surgery. BS: bariatric surgery; BMI: body mass index; TFT: thyroid function tests; US: ultrasound; TSH: thyroid-stimulating hormone; FT3: free triiodothyronine; FT4: free thyroxine; TT3: total triiodothyronine; TT4: total thyroxine; RYGB: Roux-en-Y gastric bypass; SG: sleeve gastrectomy; LSG: laparoscopic sleeve gastrectomy; TFQI: thyroid feedback quantile-based index.

Author	Study design	Population	Number of participants	Methods	Key findings
Gokosmanoglu et al. [40]	Prospective study	Male and female Adults 18-65 years undergoing BS	472	Pre and post-BS thyroid function tests (TFT) and thyroid US parameters were compared.	TSH levels decreased significantly in line with weight loss and reduction in BMI (p = 0.025) after bariatric surgery.
Yang et al. [41]	Prospective study	Male and female Adults >18 years undergoing LSG	16	FT4, FT3, and TSH parameters were retrospectively measured before and 12 months after LSG.	Mean reduction in the TSH from 2.31 to 1.54 mU/L (P=0.022). A decrease in TSH was significantly correlated with a decrease in BMI.
Lautenbach et al. [42]	Longitudinal observational study	Male and female adults ≥18 years who underwent either SG or RYGB	135	TFT parameters measured over 8 years post BS.	TSH-levels and fT3/fT4-ratio declined over the follow-up period compared to baseline (p
Liu F et al. [43]	Prospective study	Male and female adults who underwent RYGB	81	Evaluated for changes in anthropometric parameters, metabolic indexes, FT4, and TSH at baseline and 6 months after surgery.	FT4 levels decreased significantly (p <0.01 and TSH levels decreased significantly to 0.027 months post-BS
Yan et al. [44]	Longitudinal observational study	Male and female obese adults who underwent metabolic BS	470	TSH, FT3, FT4, and Thyroid Feedback Quantile-based Index (TFQI) parameters measured	TSH, fT3 levels, and TFQI were elevated at baseline and significantly decreased post-MBS.
Neves et al. [45]	Retrospective observational study	Male and female adults undergoing BS	949	Anthropometric parameters, TSH, FT4, and FT3 variation 12 months post-BS were evaluated.	Significant decrease of TSH 12 months after surgery (p<0.001).
Xia et al. [46]	Retrospective study	Male and female adults who underwent SG and RYGB.	101	TSH, FT4, FT3, Thyroid antibodies, and anthropometric parameters were measured pre and post-BS.	Mean TSH levels significantly decreased (p < 0.001), as well as a significant decrease in mean serum FT3 (p<0.001).
Karaman et al. [47]	Prospective observational study	Male and female adults who underwent LSG	106	Anthropometric parameters, serum TSH, FT3, and FT4 levels measured pre and post LSG	TSH levels decreased significantly at 1-year post op. FT3 decreased significantly at 1-month and at 1-year postoperative
Bian et al. [48]	Retrospective study	Adults >18 years following BS	287	Thyroid function parameters were measured in these patients	Serum TSH and FT3 levels decreased in obese individuals after BS
Tian et al. [49]	Prospective cohort study	Adults >18 years undergoing SG and RYGB	256	The alterations of FT4, FT3, TT4, TT3, and TSH were measured 1 year after surgery.	TSH, FT3, FT4, TT3, and TT4 all decreased significantly (P < 0.001).
Kyrou et al. [50]	Cohort study	Adults >18 years undergoing BS	10	TSH, FT4, FT3, reverse-T3, and thyroid US were performed at baseline and post-BS	No significant changes in thyroid hormone levels. Thyroid US echogenicity increased by 25% (p = 0.03)
Desousa et al. [51]	Longitudinal study	Adults 18-65 years undergoing BS	70	Thyroid US and levels of thyroid hormones were measured.	TSH levels reduced non significantly. T4 to T3 had a significant increase after BS (p = 0.01).
Chen et al. [52]	Retrospective study	Adults 18-65 years undergoing LSG	85	Changes of TSH, FT4, FT3, along with BMI were evaluated	FT3 and TSH were decreased in euthyroid patients with obesity after LSG, while FT4 was significantly increased.

Newer studies evaluating the effect of significant declines in TSH on obese patients with subclinical and overt hypothyroidism have supported previous studies [39], with an improvement in thyroid function and a decrease in the requirement of replacement levothyroxine therapy [53-58]

Pancreatic Endocrine Hormones: Insulin and Glucagon 

BS is emerging as a very effective treatment for appropriately selected patients with T2DM and obesity, and this is the most predictable and consistent endocrine change [59]. The control of diabetes is superior to lifestyle modifications and/or medical treatment, and this improvement in insulin sensitivity is mainly accounted for by enhanced secretion of insulin by pancreatic islet beta cells after being stimulated by glucagon-like peptide-1 (GLP-1) postprandially following BS [60]. The reversal of T2DM is also aided by glucagon, in addition to insulin. Multiple studies have found that glucagon secretion and peak glucagon concentrations are enhanced post-prandially following BS and are observed in both diabetic and nondiabetic controls, with the levels being higher in diabetics [61-63]. Fasting glucagon concentrations are also lower post-surgery, and these are also implicated in the significant improvement of glucose homeostasis and insulin sensitivity postoperatively [62,64,65]. GLP-1 agonist therapy is an emerging solution for glycemic control, offering comparable outcomes to BS in this area; however, BS remains superior in achieving greater reductions in BMI [66].

While the aforementioned changes in levels of insulin and glucose are desired for the improvement of insulin sensitivity in both diabetic and non-diabetic patients, they are also associated with hypoglycemia in some patients. The intestinal response of GLP-1 secretion is sometimes excessive, which then subsequently leads to a prolonged and excessive release of insulin from hypertrophied beta cells(nesidioblastosis) following a meal and a lower glucagon release, both of which then contribute to the development of asymptomatic and symptomatic hypoglycemia [67,68]. Reduced insulin clearance and increased sensitivity of beta cells to glucose following BS are other factors that contribute to high postprandial levels of insulin [68]. This is compounded by the fact that BS alters the anatomy of the gastrointestinal tract, with a reduced capacity of the stomach and uncontrolled release of food to the intestines in as much as 50% of the patients, which also contributes to the exaggerated release of insulin [69]. 

Studies have been undertaken to investigate the incidence and management of this hypoglycemia following BS. A randomized controlled trial investigated the incidence of hypoglycemia in 120 patients who underwent either SG or RYGB. Reactive hypoglycemia was detected in 29% of RYGB and 14% of SG patients, with four instances of hospitalization for symptomatic hypoglycemia only in the RYGB patients [70]. They postulated that altered beta cell sensitivity to changes in circulating levels of glucose leading to inappropriately high insulin secretion was responsible for the severity of hypoglycemia in RYGB [70]. Kim et al. found that patients with both symptomatic and asymptomatic hypoglycemia following RYGB differ minimally in plasma glucose and insulin responses following a 75 gm oral glucose tolerance test and that both these groups demonstrated hyperinsulinemia out of proportion to their insulin sensitivity as compared to obese controls [71]. Other cross-sectional analysis studies have found similar results in patients with symptomatic and asymptomatic hypoglycemia as compared to BMI-matched obese controls. They noticed the highest levels of insulin and lowest levels of glucose in patients with neuroglycopenia, highlighting the role of insulin [72,73]. They also found that glucagon and GLP-1 were elevated post-prandially, with GLP-1 especially high in patients with neuroglycopenia [73]. Salehi et al. have shown that blockades of GLP-1 receptors can successfully correct hypoglycemia and are promising therapeutic targets [74]. 

This hyperinsulinemic hypoglycemia might occur late after surgery, as reported by a case series, occurring 15 to 37 months after RYGB [75]. It is not limited to SG and RYGB and has been noted in follow-ups with several patients after LAGB [76]. 

**Figure 2:**
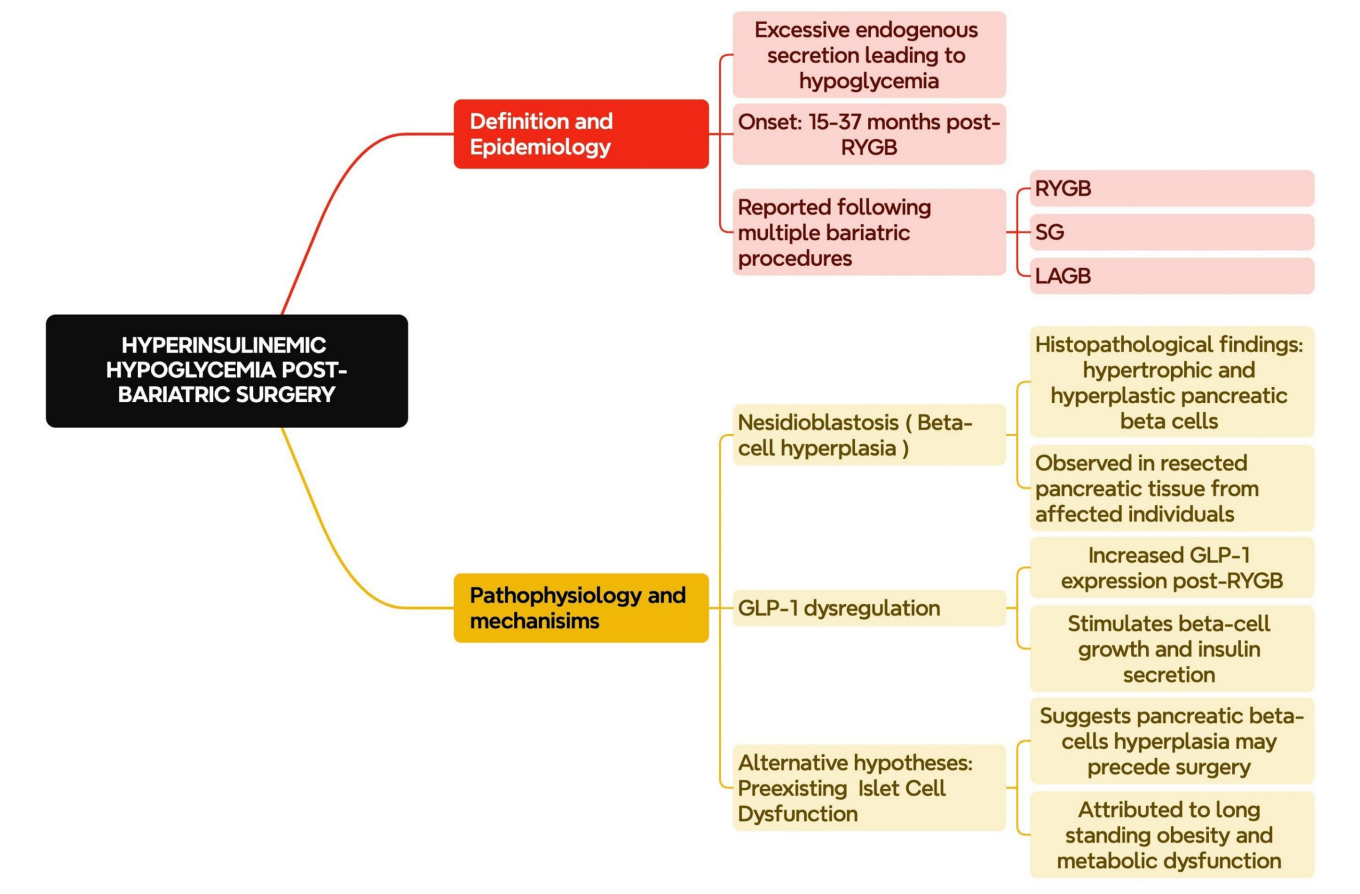
Hyperinsulinemic hypoglycemia post-bariatric surgery. The figure is created by the authors. RYGB: roux-en-Y gastric bypass; SG: sleeve gastrectomy; LAGB: laparoscopic adjustable gastric banding; GLP-1: glucagon-like peptide-1.

Hypertrophy of pancreatic beta cells, also known as nesidioblastosis, is a major finding in cases of hyperinsulinemic hypoglycemia. Multiple studies have demonstrated the development of nesidioblastosis following BS after resection and histopathological examination of pancreatic tissue in an effort to manage troublesome recurrent hypoglycemia. Vella et al. examined the resected pancreatic tissue of 23 patients with severe hyperinsulinemic hypoglycemia, which had nesidioblastosis in most specimens, although some had functioning insulinomas [77]. Other studies have demonstrated nesidioblastosis in patients requiring partial pancreatectomy for neuroglycopenic hypoglycemia post-RYGB, which could not be managed by dietary modifications [78,79]. While all these authors agree that RYGB leading to enhanced expression of GLP-1 is the cause of this hypertrophy and subsequent hypoglycemia, some authors have controversially suggested that this hypertrophy exists preoperatively and is attributed to long-standing obesity (Figure 2) [80,81].

Variations in pancreatic and other related hormones following bariatric surgery are highlighted in Table 3.

**Table 3:** Variations in pancreatic and related hormones following bariatric surgery RYGB: roux-en-Y gastric bypass; SG: sleeve gastrectomy; SX: symptomatic; ASX: asymptomatic; GB: gastric bypass; GLP-1: glucagon-like peptide-1; Ex-9: GLP-1 receptor antagonist; LAGB: laparoscopic adjustable gastric banding; STM: standardized test meals; GIP: glucose-dependent insulinotropic polypeptide; PDX-1: pancreatic and duodenal homeobox 1; NG: neuroglycopenia.

Study	Study design	Population	Number of participants	Methods	Key findings
Capristo et al. [70]	Randomized open-label trial	Patients were randomized 1:1 to RYGB or SG	120	Incidence of reactive hypoglycemia at 1 year after surgery along with insulin sensitivity andsecretion and lipid profile.	Reactive hypoglycemia was detected following both SG and RYGB (p=0.079). β-cell glucose sensitivity index increased after both treatments (p<0.001).
Kim et al. [71]	Prospective cohort study	Symptomatic(SX) hypoglycemia and asymptomatic(ASX) patients following RYGB.	108	Plasma glucose and insulin concentrations were measured after a 75-gmoral glucose challenge in five groups.	SX-RYGB group had higher 30-min glucose after oral glucose compared with the ASX-RYGB group (p=0.04). Peak insulin concentrations post-RYGB were increased.
Salehi et al. [72]	Cross-sectional analysis	Adults who underwent GB	76	Analysis of insulin secretion rate and islet, as well as gastrointestinal hormone responses to liquid mixed meal ingestion by clinical history.	Hypoglycemic GB subjects had lower postprandial insulin clearance rates and higher insulin secretion rates during the glucose decline after the test meal.
Goldfine et al. [73]	Cross-sectional analysis	Adults with symptomatic hypoglycemia (NG) and asymptomatic adults after RYGB surgery	36	Analysis of metabolic variables in both the fasting state and after a liquid mixed-meal challenge.	Insulin & C-peptide after the meal were both higher NG vs asymptomatic individuals. Glucagon and GLP-1 levels were higher in both post-GB groups & GLP-1 was markedly higher in the group with NG.
Salehi et al. [74]	Cross-sectional study	Adults who underwent RYGB	24	Underwent a mixed-meal tolerance test and received a continuous infusion of the GLP-1 receptor antagonist (Ex-9). Glucose kinetics, as well as islet and gut hormone responses, were measured.	Infusion of Ex-9 corrected hypoglycemia in all patients with HGB & the reduction in postprandial insulin secretion by Ex-9 was greater in the H-GB group than in the other groups (p<0.05).
Bantle et al. [75]	Cross-sectional study	Male and female adults who underwent RYGB	3	Response to high and low carbohydrate test meals.	Demonstrated peak plasma glucose >200 mg/dl and peak serum insulin >300 microU/l. Despite rapid serum insulin decline, all three developed hypoglycemia following low carbohydrate test meals.
Scavini et al. [76]	Prospective cohort study	Male and female adults who underwent LAGB	221	Measured serum levels of glucose and insulin after administration of 75 g of glucose before surgery and after surgery.	During follow-up, nine episodes of asymptomatic hyperinsulinemic hypoglycemia were recorded in 8 patients.
Service et al. [78]	Retrospective study	Adults who underwent RYGB	6	Glucose, insulin, C-peptide, sulfonylurea levels, and pancreatic histopathology were evaluated.	Serum insulin and C-peptide were elevated, while serum glucose decreased during hypoglycemic episodes. Nesidioblastosis identified in histopathology.
Rabiee et al. [79]	Prospective study	Patients with recurrent neuroglycopenia following RYGB	9	Standardized test meals (STM) to evaluate glucose, insulin, and incretin (GLP-1, GIP) responses and hyperglycemic clamps to assess beta-cell function. A patient underwent a distal pancreatectomy.	Elevated GLP-1, insulin, and GIP levels during STM. Octreotide reduced GLP-1 and insulin response. Histology revealed enlarged islets, ectopic β-cells, and increased PDX-1.
Patti et al. [80]	Retrospective study	Adults who underwent RYGB	3	Glucose, insulin, and C-peptide levels in fasting and postprandial states and Pancreatic histopathology	Evidence of severe postprandial hyperinsulinemia and hypoglycemia. Pathology in all revealed diffuse islet hyperplasia and expansion of beta cell mass.
Meier et al. [81]	Retrospective study	Adults who underwent RYGB	53	The study evaluated the pancreas of patients post RYGB with hypoglycemia compared to controls.	In patients with post-GB hypoglycemia, beta-cell nuclear diameter was increased (p<0.001).

The counter-regulatory responses to hypoglycemia are also blunted following BS. Mathur et al. described reversible adrenal insufficiency in three patients, which was due to attenuation of counterregulatory responses to hypoglycemia following hyperinsulinemic hypoglycemia post-BS, which consequently suppressed the hypothalamic-pituitary-adrenal (HPA) axis [82]. Tripyla et al. found significantly lower levels of glucagon during post-bariatric hypoglycemia in a study of 32 patients [83]. Abrahamsson et al. found that there were markedly low levels of counter-regulatory hormones glucagon, cortisol, and catecholamines and reduced sympathetic nerve responses in post-bariatric hypoglycemia [84]. GH had a delayed response but reached an overall higher peak [84]. Thus, the counter-regulatory mechanisms for hypoglycemia are also attenuated in hyperinsulinemic hypoglycemia following BS.

Insulin, along with gut hormones like ghrelin, leptin, gastrin, GLP-1, and cholecystokinin (CCK), acts on the vagus nerve [85]. This modulation of the vagus nerve due to altered levels of hormones leads to control of appetite, satiety, and absorption of nutrients and is implicated in weight loss [85-87]. The increase of insulin and these other hormones has now been correlated to the development of an exaggerated vagal tone and pathological vasovagal syndromes following BS with presenting symptoms of lightheadedness, presyncope, and syncope. Many studies have now documented this incidence of new orthostatic intolerance following BS, which includes [88-92]

In summary, bariatric surgery significantly improves insulin sensitivity and T2DM through hormonal changes like enhanced GLP-1 secretion and improved glucose homeostasis. However, these benefits can lead to complications such as hypoglycemia, nesidioblastosis, and altered counter-regulatory responses. The modulation of vagal tone may also cause orthostatic intolerance. Addressing these challenges requires tailored management and further research to balance the surgery’s metabolic benefits with its risks.

Cortisol

BS alters the body’s counter-regulatory response to hypoglycemia, which includes the hormone cortisol, among others. Fanni et al. investigated the brain connectivity responses to hypoglycemia before and 4 months following RYGB in 10 patients. They noted altered connectivity of several brain pathways and significantly reduced Adrenocorticotropic Hormone (ACTH) and cortisol responses during hypoglycemia [93]. Abrahamsson et al. examined 12 obese patients without T2DM before and 23 weeks following RYGB and documented markedly reduced cortisol, along with glucagon and catecholamines, during hypoglycemia [84]. 

Parathyroid

Systematic reviews and meta-analyses have established the effect of BS on parathyroid hormone (PTH). BS is associated with a significantly increased risk of secondary hyperparathyroidism [94]. The increase in PTH is greater in RYGB compared to SG, and both are associated with significantly increased bone turnover markers, which enhance skeletal fragility over time [95,96].

Clinical implications and limitations

The hormonal changes following BS have profound implications on patient health, with both beneficial and adverse outcomes. While improvements in the somatotropic axis, gonadal hormones, and pancreatic function correlate with enhanced metabolic control and fertility outcomes, other changes, such as persistent IGF-1 suppression, hyperinsulinemic hypoglycemia, and bone demineralization, reveal significant dysfunction. Despite these correlations, the clinical impact of many hormonal shifts, such as reduced TSH in euthyroid patients or altered counter-regulatory responses to hypoglycemia, remains unclear. Much of the evidence is derived from association studies with limited causality, underscoring the need for research that integrates biochemical findings with clinical presentations.

Future Challenges

Future research must prioritize high-quality longitudinal studies to elucidate the causal links between BS and endocrine dysfunction. Understanding the mechanisms underlying persistent hormonal imbalances, such as the dissociation of GH and IGF-1 restoration or long-term catabolic states, will be critical. Investigating therapeutic interventions, including hormone replacement or modulators of gut hormones like GLP-1 receptor blockers, could mitigate adverse effects while preserving the surgery’s metabolic benefits. These efforts will also require expanding study populations and incorporating diverse demographic and surgical subtypes to ensure generalizable findings that guide patient-specific care.

## Conclusions

BS represents a dual-edged sword in endocrine health, offering remarkable benefits like improved glycemic control and fertility while predisposing patients to hormonal dysfunctions such as secondary hyperparathyroidism, bone loss, and hypoglycemia. Bridging research gaps through focused, mechanism-driven studies will be essential to balancing these effects and optimizing surgical outcomes. A holistic approach that integrates preoperative screening, tailored postoperative care, and novel therapeutic strategies is crucial to ensuring that BS achieves sustainable benefits for patients.
